# Fracture Resistance of Zirconia Oral Implants In Vitro: A Systematic Review and Meta-Analysis

**DOI:** 10.3390/ma13030562

**Published:** 2020-01-24

**Authors:** Annalena Bethke, Stefano Pieralli, Ralf-Joachim Kohal, Felix Burkhardt, Manja von Stein-Lausnitz, Kirstin Vach, Benedikt Christopher Spies

**Affiliations:** 1 Department of Prosthodontics, Geriatric Dentistry and Craniomandibular Disorders, Charité—Universitätsmedizin Berlin, corporate member of Freie Universität Berlin, Humboldt-Universität zu Berlin, and Berlin Institute of Health, Aßmannshauser Str. 4-6, 14197 Berlin, Germany; a.k.bethke@web.de (A.B.); stefano.pieralli@charite.de (S.P.); felix.burkhardt@charite.de (F.B.); manja.von-stein-lausnitz@charite.de (M.v.S.-L.); 2 Department of Prosthetic Dentistry, Faculty of Medicine, Center for Dental Medicine, Medical Center—University of Freiburg, Hugstetter Str. 55, 79106 Freiburg, Germany; ralf.kohal@uniklinik-freiburg.de; 3 Institute of Medical Biometry and Statistics, Faculty of Medicine, Medical Center—University of Freiburg, University of Freiburg, Stefan-Meier-Str. 26, 79104 Freiburg, Germany; kv@imbi.uni-freiburg.de

**Keywords:** dental implant, zirconia, ceramics, aging, artificial mouth, fracture load, fatigue, chewing simulation, meta-analysis

## Abstract

Various protocols are available to preclinically assess the fracture resistance of zirconia oral implants. The objective of the present review was to determine the impact of different treatments (dynamic loading, hydrothermal aging) and implant features (e.g., material, design or manufacturing) on the fracture resistance of zirconia implants. An electronic screening of two databases (MEDLINE/Pubmed, Embase) was performed. Investigations including > 5 screw-shaped implants providing information to calculate the bending moment at the time point of static loading to fracture were considered. Data was extracted and meta-analyses were conducted using multilevel mixed-effects generalized linear models (GLMs). The Šidák method was used to correct for multiple testing. The initial search resulted in 1864 articles, and finally 19 investigations loading 731 zirconia implants to fracture were analyzed. In general, fracture resistance was affected by the implant design (1-piece > 2-piece, *p* = 0.004), material (alumina-toughened zirconia/ATZ > yttria-stabilized tetragonal zirconia polycrystal/Y-TZP, *p* = 0.002) and abutment preparation (untouched > modified/grinded, *p* < 0.001). In case of 2-piece implants, the amount of dynamic loading cycles prior to static loading (*p* < 0.001) or anatomical crown supply (*p* < 0.001) negatively affected the outcome. No impact was found for hydrothermal aging. Heterogeneous findings of the present review highlight the importance of thoroughly and individually evaluating the fracture resistance of every zirconia implant system prior to market release.

## 1. Introduction

To date, titanium can be considered the gold standard material in oral implantology [1]. However, due to increasing esthetic standards and a discussed impact of metal/titanium particle release on the pathogenesis of peri-implant bone loss [2,3], a renaissance of ceramic oral implants can be observed in dental media. Nowadays, the market share of zirconia oral implants seems to be increasing, even if still comparatively small compared to conventional titanium implants.

Nonetheless, the superiority of ceramic oral implants regarding esthetics and biocompatibility, or, as an example, the frequently claimed patients’ demand for metal-free implantology are still not soundly scientifically evidenced. Nevertheless, the majority of dental experts are of the opinion that zirconia oral implants will be coexistent with titanium implants in the near future [4].

When zirconium dioxide (zirconia, ZrO_2_) was introduced as ceramic implant material, research focused to evaluate and improve its osseointegrative potential by creating a microroughened surface topography [5]. In the first instance, parameters like bone-to-implant contact (BIC), push-in values and removal torque were assessed in animal experiments. As a result, zirconia implants with various surface modifications (additive by sintering a porous ceramic layer, subtractive by sandblasting and/or acid-etching or, for example, by texturing the inner surface of a mold in case of an injection-molded implant) can nowadays be considered comparable to titanium implants by means of osseointegration in preclinical studies [6]. This finding was confirmed in clinical trials, however limited to short- and mid-term observation periods and the replacement of up to three adjacent missing teeth (single-tooth restorations and three-unit fixed dental prostheses) using one-piece ceramic implants [7].

From a technical point of view, such a 1-piece design, comprising the abutment and endosseous part in a single piece, might benefit from increased fracture resistance and reduced susceptibility for low-temperature degradation or so-called “aging” (by exposing a reduced total surface area to aging by inducing oral fluids), compared to 2-piece ceramic implants. Furthermore, 1-piece implants do not have a micro-gap in between the assembled implant and abutment. One might consider the absence of such a micro-gap beneficial, since it is capable in hosting bacteria, potentially resulting in marginal inflammation and consecutive bone resorption [8]. However, no advantage of a monobloc design was found for “seamless”, 1-piece implants made from titanium [9]. Moreover, from a practitioner’s point of view, a 1-piece implant design is associated with several surgical and prosthodontic shortcomings [10]. As an example, submerged implant healing is hardly possible, since the transmucosal part of a 1-piece implant cannot be detached. If no sufficient primary stability can be attained or guided bone regeneration is necessary, a missing option for wound closure might be considered disadvantageous. Furthermore, there is only a limited potential to compensate for mal-positioned implants with the provisional and final restoration. When trying to remove subsections in case of misaligned implants to support a bridge, intra-oral grinding of the zirconia abutment is necessary [11]. This, however, might have an impact upon the osseointegration (due to potential heat development or the displacement of zirconia particles in surrounding tissues) and fracture resistance of the implant [12]. Therefore, a two-piece design represents the favorable option for daily clinical use. Today, several two-piece zirconia implants are available on the market. In these systems, implant-abutment assembly is mostly realized by either luting the abutment to the implant or by screw-retention [13]. Luting the abutment to the implant seals the micro-gap, and allows for initial but irreversible correction of the implant angulation, but misses flexibility for future restorations of the implant. On the other hand, when going for screw-retention, several ceramic implants are still assembled with a titanium screw, and therefore, still not metal-free in the proper sense.

Even if the market share of zirconia dental implants increases, concerns regarding their fracture resistance are still present, and standardized testing protocols for zirconia implants adequately addressing the aging behavior of the final product are still missing [14]. To overcome this, different treatments were proposed to mimic intraoral conditions to the extent possible for the evaluation of ceramic implants. These treatments included thermal aging (high-temperature conditions or thermal cycling) [15,16] and/or dynamic loading procedures (various exposure times and different applied loading modes) [12,17]. Zirconia implants evaluated regarding their fracture resistance in the literature comprised a heterogeneous range of features like material selection (yttria-stabilized tetragonal zirconia polycrystal, Y-TZP or alumina-toughened zirconia, ATZ) [18], design (1- or 2-piece) [13], manufacturing (subtractive or by ceramic injection molding, CIM) [19], restoration (anatomical crown, hemisphere or no restoration) [20,21], abutment preparation (in the case of 1-piece implants) [22], or assembly (in the case of 2-piece implants) [13].

Therefore, the objective of the present systematic review was to evaluate the influence of the aforementioned treatments and features on the fracture resistance of zirconia oral implants in different preclinical studies. The null hypothesis supposed no distinction between treatments and features in relation to bending moment when statically loading the implant to fracture.

## 2. Materials and Methods

### 2.1. Study Design

To determine a selection of comparable studies on the question of zirconia implant fracture resistance, the preferred reporting items for systematic reviews and meta-analyses (PRISMA) statement of 2009 was applied [23]. Therefore, this report takes the appropriate Enhancing the Quality and Transparency of health Research (EQUATOR) (http://www.equator-network.org) guidelines into account.

### 2.2. Focused Question

Is there a variable significantly affecting the fracture resistance of 1- and 2-piece zirconia implants in preclinical in-vitro studies?

### 2.3. Search Strategy

Two databases, namely the Medical Literature Analysis and Retrieval System Online (MEDLINE) (PubMed) and Embase (accessed via Ovid), were screened for relevant articles. The database specific search strategies consisted of a combination of subject headings and free text words. Data was extracted from the databases on 3rd December 2019 without applying any time restrictions. Thereafter, references of included articles were screened for further records satisfying the inclusion criteria (cross-referencing). In case of the availability of the full methodological procedures in the literature and accessibility of information regarding the included samples, unpublished data of the authors of the present review was likewise included. The resulting studies were imported and stored in a reference managing program (EndNote X9; Clarivate Analytics, Philadelphia, PA, USA). Articles written in English and the German language were considered.

### 2.4. Screening Process

To build up the search terms, three categories addressing the samples (dental implants), materials (zirconia ceramics) and outcome (fracture load) were combined (“AND”). These categories consisted of combinations (“OR”) of free text words and indexed vocabulary (MEDLINE: MeSH terms, Embase: Emtree terms). An asterisk was used in combination with some free text words as a truncation symbol (e. g. “ceramic *”) to allow for the so-called “wildcard search”.

Pubmed search term:
((((dental implant [MeSH Terms]) OR ((((oral) AND ((implant) OR implants))) OR ((dental) AND ((implant) OR implants))))) AND (((zircon *) OR ceramic *) OR ceramics[MeSH Terms])) AND (((((ageing) OR aging) OR artificial mouth) OR fracture resistance) OR load *)

Embase search term:
(‘tooth implant’/exp OR (oral AND implant) OR (dental AND implant)) AND (zircon * OR ceramic * OR ‘ceramics’/exp) AND (ageing OR aging OR (artificial AND mouth) OR (fracture AND resistance) OR load *)

### 2.5. Eligibility Criteria

Studies to be included in this systematic review needed to fulfill the following inclusion criteria:

- Language: English or German
- Samples: Screw-shaped, ceramic oral implants containing a minimum of 50% *v*/*v* ZrO_2_ within the bulk material
- Outcome: Static loading to fracture
- Outcome measure: Bending moment [Ncm or Nmm] or fracture load [N] allowing to calculate the bending moment (e.g., by adopting ISO 14801 or providing data to calculate the lever arm) was provided
- Sample size: Minimum of five samples tested

### 2.6. Selection of Studies

Concerning the inclusion criteria, both the first author and the senior author of this manuscript (A.B. and B.C.S.) independently screened the titles and abstracts of the extracted data in the reference management program. If sufficient information needed for inclusion or exclusion was not provided within the title or abstract, the corresponding full texts were read. In case of disagreement, a third author (S.P.) was consulted for final decision making.

### 2.7. Data Extraction

Besides the total number of samples within one study, the number of implants made from different materials (Y-TZP, ATZ), processing routes (subtractive, injection molding), design (1- and 2-piece) and diameters were retrieved. Further features like restoration mode (anatomical crown, hemisphere or no reconstruction), abutment preparation (yes/no in case of 1-piece implants), implant-abutment connection (screwed/bonded in case of 2-piece implants), thermal aging (thermal cycling, high temperature, no aging) or dynamic loading (yes/no), dynamic loading conditions (exerted load and amount of cycles), crosshead speed during static fracture, and angulation, were likewise extracted. This allowed us to group the implants finally subjected to static loading within the included studies in cohorts. For standardization purposes, the bending moment at the time point of fracture [Ncm] was considered the outcome measure of interest, and the corresponding authors of the articles to be included were contacted by email in case of solely providing fracture load values [N] without mentioning the lever arm. Extracted cohorts were subdivided into groups subjected to comparable treatments:

1. No dynamic loading
2. 1–1.2 million loading cycles (50 N)
3. 1–1.2 million loading cycles (100 N)
4. 3.5–5 million loading cycles (100 N)
5. 5 million loading cycles (>500 N)
6. 10 million loading cycles (100 N)

### 2.8. Statistical Analysis

From the included nineteen studies/datasets, two to twelve observations were extracted each. One observation consisted of the mean bending moment and standard deviation (at the time point of fracture) and/or mean fracture load and standard deviation (including additional information allowing us to calculate the bending moment) of a specific cohort of implants (comprising the same type of implant subjected to the same treatment) extracted from one included study. These observations had sample sizes of 2 to 12 implants. To analyze the effect of specific treatments of features (as indicated in 2.7) on the bending moment, a multilevel mixed-effects generalized linear model was used for each outcome, with each investigation as random effect to cluster observations by the respective studies. The Šidák method was used to correct for multiple testing. The level of significance was set at *p* < 0.05.

In order to compare the aforementioned groups (1–6, depending on load and cycles) for heterogeneity of the data, both inter- and intra-standard deviations with 95% confidence intervals (Cis) were computed. In addition, the cohort-specific standard error of the bending moment was used for weighting. Furthermore, box plots were created for visualization of the data. The data were analyzed with STATA 16.1 (StataCorp LLC, Texas, TX, USA).

## 3. Results

### 3.1. Screening Process/Included Data

**Figure 1:**
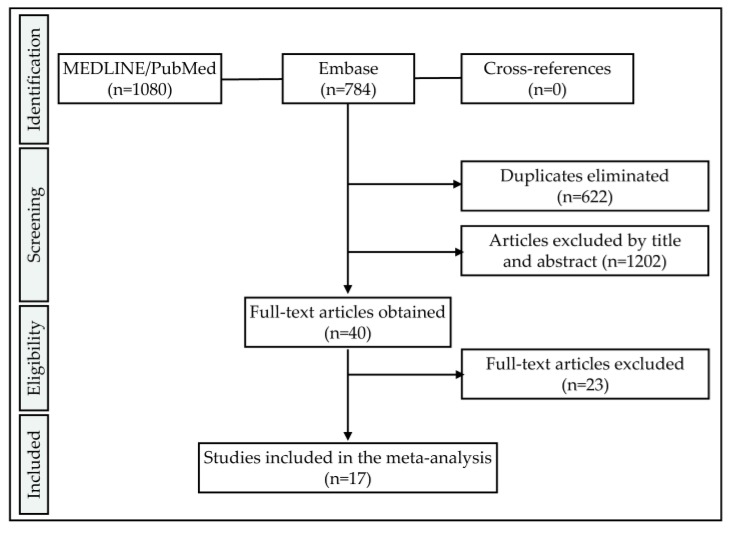
Flowchart according to the preferred reporting items for systematic reviews and meta-analyses (PRISMA) guidelines.

Screening of two databases using the aforementioned specifically adapted search terms resulted in a total of 1864 records. After the removal of 622 duplicates, another 1202 records were withdrawn for analyses by screening the titles and abstracts. After reading the full texts of the remaining 40 studies, a further 23 manuscripts were excluded (Figure 1). Detailed reasons for exclusion can be found in Table A1. In general, the most frequent reasons for exclusion were the fracture of zirconia abutments assembled with titanium implants (mostly excluded by title and abstract) and the fracture on the restoration level using zirconia one-piece implants as support (mostly excluded during full-text screening). When only the fracture load [N] during static loading was reported, three options allowed for the calculation of the bending moment: (1) embedding was described to fully respect ISO 14801 (prescribing a lever arm of 5.5 mm allowing for the calculation of the bending moment), (2) all details regarding the embedding were provided in the manuscript (e.g., by providing a scheme) or (3) the bending moment and/or lever arm were provided by the authors upon request. As an example, six of the included studies adopted ISO 14801 for embedding [15,17,21,24,25,26], whereas three provided all necessary information [19,27,28] allowing us to calculate the bending moment (embedding level, angulation, total sample length, point of loading). In the remaining cases the bending moment was reported [13,20,22] or sent by the authors [12,18,29,30]. Finally, 17 full-texts were analyzed in the present systematic review (Table 1). In addition, the datasets of two finalized projects, currently under review and in preparation of the manuscript, were included. Two authors of the present review (R.K. and B.C.S.) were involved in both of these two investigations, and were able to access the full data. The applied materials and methods were already described in detail in precedent publications [21,26]. Since available on the market, the material composition of the included implant systems is likewise available and accessible. In detail, three zirconia implant systems (1-piece: Straumann PURE Ceramic, Straumann AG, Basel, CH; 2-piece: 5s-50-10, Z-Systems AG, Oensingen, CH and Ceralog Hexalobe Implant, Axis biodental, Les Bois, CH) were subjected to identical treatments and fracture load measurements, as described in two of the included studies [21,26]. In the case of Straumann 1-piece (as-received: 609 ± 20 Ncm; loaded/aged: 557 ± 36 Ncm) and Z-Systems 2-piece implants (as-received: 463 ± 21 Ncm; loaded/aged: 443 ± 39 Ncm), aging/loading (as described in [21,26]) did not affect the fracture resistance to a statistically significant level (*p* = 0.171). In contrast, the fracture resistance of 2-piece Ceralog Hexalobe Implants (as-received: 547 ± 89 Ncm; loaded/aged: 413 ± 127 Ncm) was significantly affected (*p* = 0.046) by aging/loading (as described in [21,26]).

### 3.2. Meta-Analyses

All 17 articles published between 2009 [29] and 2019 [15,16] were included and analyzed in the present meta-analysis. Moreover, unpublished data of two projects currently under review and in preparation of the manuscript were included (Table 1). From the included articles/datasets, 114 observations were extracted or calculated (mean bending moment), comprising different implant features (e.g., diameter, material, crown supply, abutment preparation or implant-abutment-connection) or treatments (e.g., thermal aging or dynamic loading). One observation consisted of the mean bending moment and standard deviation (SD) of up to 12 included implants.

In order to evaluate the impact of different dynamic loading procedures (implants were subjected to prior to fracture loading) on the outcome (bending moment), groups as indicated in Section 2.7 were analyzed for heterogeneity. As a result, standard deviation as a measure of variation within and in between the included studies revealed to be within the same range (Table 2). No heterogeneity of the bending moments for groups 1–6 was found, even if a decreased mean value for group 3 was calculated (*p* = 0.612). This did not change when stratifying the implants according to their design (1-piece: *p* = 0.951; 2-piece: *p* = 0.056).

### 3.3. Outcomes

Outcomes extracted from the 17 included studies and the two unpublished datasets were calculated and stratified for the material selection, manufacturing, implant diameter, anatomical crown supply, abutment preparation (1-piece implants), implant-abutment-connection (IAC; 2-piece implants), thermal aging procedure prior to static loading (none; TC = thermal cycling, mostly in between 5–55 °C; HT = high temperature, mostly in between 60–134 °C) and/or dynamic loading in a chewing simulation device applying different loads (ranging from 50 to > 500 N) for a different amount of cycles (ranging from 1 to 10 millions). In total, 731 implants were available for analyses, revealing a mean bending moment at the time point of fracture of 386.4 ± 167.6 Ncm. Furthermore, the outcome was stratified for 1- and 2-piece implants. Mean bending moments, standard deviations and the included number of implants are listed in Table 3. Significance (linear mixed models, level of significance *p* < 0.05) calculated for differences regarding the implant design, different covariables and treatments can be found in Table 4.

#### 3.3.1. Implant Design

**Figure 2:**
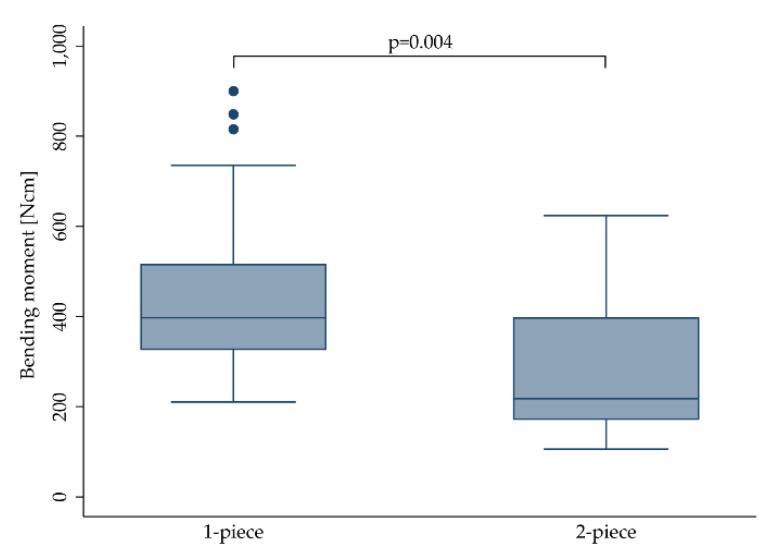
Boxplot showing the bending moment at the time point of fracture for 1- and 2-piece zirconia implants. Whiskers are used to represent all samples lying within 1.5 times the interquartile range (IQR). Dots represent outliers. Detailed data can be found in Table 3 and Table 4.

Eight studies [12,15,17,18,20,22,25,29] focused on 1-piece zirconia implants, whereas six studies solely included 2-piece implants [16,19,21,24,26,30]. The remaining investigations evaluated a mixture of both 1- and 2-piece implants [13,27,28]. Regardless of all other variables, 1-piece implants (431.9 ± 151.0 Ncm) were found to be more fracture resistant than 2-piece implants (291.7 ± 162.4 Ncm, *p* = 0.004; Figure 2).

#### 3.3.2. Material

**Figure 3:**
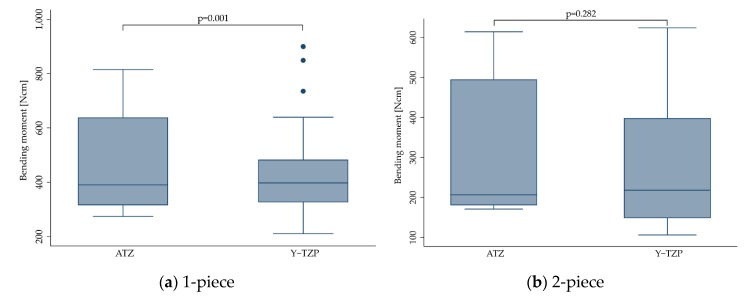
Boxplots showing the bending moment at the time point of fracture depending on the material selection for 1- (**a**) and 2-piece (**b**) zirconia implants. Whiskers are used to represent all samples lying within 1.5 times the interquartile range. Dots represent outliers. Detailed data can be found in Table 3 and Table 4.

Material selection of the included studies is listed in Table 1. Of the included implants, 577 were made from Y-TZP, whereas 154 were manufactured from ATZ [13,18,19,22,24,26]. When pooling the outcome for 1- and 2-piece zirconia implants, the bending moment at the time point of implant fracture was significantly affected by the material (*p* = 0.002; Table 4). In detail, implants made from alumina-toughened zirconia (ATZ, 418.7 ± 106.0 Ncm) were more fracture-resistant compared to implants made from yttria-stabilized tetragonal zirconia polycrystals (Y-TZP, 378.7 ± 160.1 Ncm, *p* = 0.002). When stratifying the outcome for 1- and 2-piece implants, however, material selection only affected 1-piece implants (*p* = 0.001, Figure 3a), whereas 2-piece implants performed the same, regardless of the material selection (*p* = 0.282, Figure 3b).

#### 3.3.3. Manufacturing

**Figure 4:**
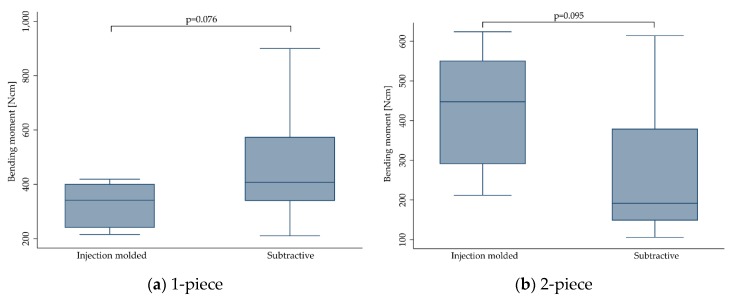
Boxplots showing the bending moment at the time point of fracture depending on the manufacturing method for 1- (**a**) and 2-piece (**b**) zirconia implants. Whiskers are used to represent all samples lying within 1.5 times the interquartile range. Detailed data can be found in Table 3 and Table 4.

Manufacturing was mostly subtractive (*n* = 591 implants), but ceramic injection-molding (CIM) was likewise used for the production (*n* = 120 implants) [15,19,21,25]. There was no statistically significant difference in the fracture resistance of implants when manufacturing method (subtractive: 397.5 ± 177.4 Ncm, CIM: 364.8 ± 116.7 Ncm) was regarded (*p* > 0.095). Boxplots can be seen in Figure 4.

#### 3.3.4. Implant Diameter

**Figure 5:**
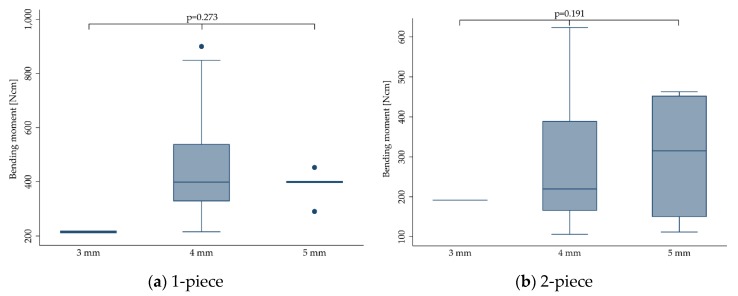
Boxplots showing the bending moment at the time point of fracture depending on the implant diameter for 1- (**a**) and 2-piece (**b**) zirconia implants. Whiskers are used to represent all samples lying within 1.5 times the interquartile range. Dots represent outliers. Detailed data can be found in Table 3 and Table 4.

No statistically significant difference could be calculated for the bending moment at the time point of fracture regarding the implant diameter ranging from 3 to 5 mm (*p* = 0.327). This did not change when stratifying the outcome for 1- (*p* = 0.273) and 2-piece (*p* = 0.191) implants. However, the included studies evaluated only very few implants in the range of 3 mm (range: 3.0–3.3 mm; *n* = 15, 207.2 ± 14.3 Ncm) [24,27] and 5 mm (range: 4.5–5.0 mm; *n* = 41, 349.4 ± 125.4 Ncm) [15,19,27,28], whereas the majority of implants had a diameter in the range of 4 mm (range: 3.8–4.4 mm; *n* = 675, 394.9 ± 170.4 Ncm). Boxplots can be seen in Figure 5.

#### 3.3.5. Anatomical Crown Supply

**Figure 6:**
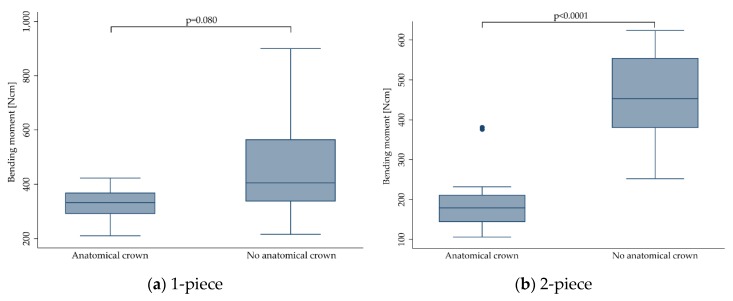
Boxplots showing the bending moment at the time point of fracture depending on the crown supply for 1- (**a**) and 2-piece (**b**) zirconia implants. Whiskers are used to represent all samples lying within 1.5 times the interquartile range. Dots represent outliers. Detailed data can be found in Table 3 and Table 4.

Of the included 731 implants, 209 were restored with an anatomically shaped crown, mostly made from ceramic materials. Most of the crowns were designed to replace maxillary central incisors but also some premolar reconstructions were included. The remaining 522 implants did not receive any reconstruction and were directly loaded to the abutment or were equipped with a non-anatomical stainless-steel hemisphere according to ISO 14801. When pooling the data for 1- and 2-piece implants, anatomical crown supply (237.5 ± 96.6 Ncm) negatively affected the outcome compared to implants with no crowns or equipped with a hemisphere (455.2 ± 147.7 Ncm, *p* < 0.0001). When stratifying for 1- and 2-piece implants (Figure 6), statistical significance was only reached for the group of 2-piece implants (*p* < 0.0001), likewise revealing an inferior outcome for implants restored with anatomical crowns. Fracture resistance of 1-piece implants was not affected by crown supply (*p* = 0.080).

#### 3.3.6. Abutment Preparation and Implant-Abutment-Connection (IAC)

**Figure 7:**
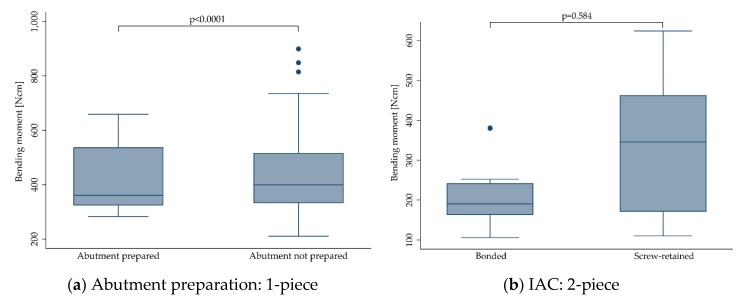
Boxplots showing the bending moment at the time point of fracture depending on the abutment preparation for 1-piece (**a**) and depending on the implant-abutment-connection (IAC) for 2-piece (**b**) zirconia implants. Whiskers are used to represent all samples lying within 1.5 times the interquartile range. Dots represent outliers. Detailed data can be found in Table 3 and Table 4.

Of the 1-piece implants (*n* = 495), 112 abutments were prepared/modified by grinding [12,18,22,29], whereas 383 abutments remained untouched until fracture. In most cases, abutment preparation should simulate a clinically relevant situation of a 1-piece implant installed in anterior regions of the mouth. In both groups, some implants were restored with anatomically shaped incisor crowns, and some did not receive any reconstruction. Grinding of the abutment (411.3 ± 126.2 Ncm) resulted in a significantly reduced bending moment at the time point of fracture compared to non-grinded implants (436.5 ± 156.5 Ncm, *p* < 0.0001; Figure 7a).

Of the two-piece implants included in the present review (*n* = 236), 159 abutments were assembled by screw retention [13,16,19,21,24,26]. Most screws were made from titanium, but also gold and polyetheretherketone (PEEK; in one study, carbon-fiber-reinforced [26]) were used. The remaining 77 two-piece implants were irreversibly assembled by adhesive bonding [13,19,27,28,30]. The type of abutment retention (screw-retained: 327.5 ± 179.0 Ncm, bonded: 217.0 ± 86.0 Ncm) did not affect the fracture resistance (*p* = 0.584; Figure 7b).

#### 3.3.7. Thermal Aging

**Figure 8:**
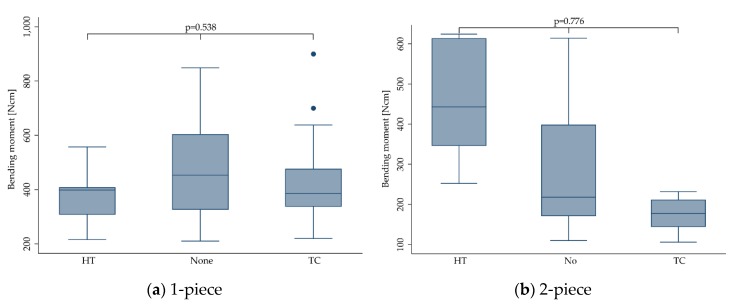
Boxplots showing the bending moment at the time point of fracture, depending on the thermal aging conditions (none, HT = high temperature, TC = thermal cycling) for 1- (**a**) and 2-piece (**b**) zirconia implants. Whiskers are used to represent all samples lying within 1.5 times the interquartile range. Dots represent outliers. Detailed data can be found in Table 3 and Table 4.

Regardless of the implant design, in 297 implants, no aging was induced prior to static loading to fracture, whereas 124 implants were subjected to a high temperature (HT) treatment in a humid environment, ranging from 60 up to 134 °C for different time periods lasting from 5–30 h (134 °C) [15,25] to 60 days (85 °C) [21,26]. High temperature treatment was applied in combination or during dynamic loading or alone. The remaining 310 implants were subjected to a thermal cycling (TC) procedure, exposing the samples to a changing water bath set at 5 and 55 °C [12,16,18,19,20,22,27,28,29,30]. The latter was mostly performed during dynamic loading in a chewing simulation device. Compared to untreated implants (406.2 ± 180.4 Ncm), neither HT treatment (392.9 ± 115.9 Ncm) nor TC (355.5 ± 171.7 Ncm) did affect the fracture resistance (*p* = 0.446). This did not change when calculating the outcome for 1- (*p* = 0.538) and 2-piece implants (*p* = 0.776) separately (Figure 8).

#### 3.3.8. Dynamic Loading

**Figure 9:**
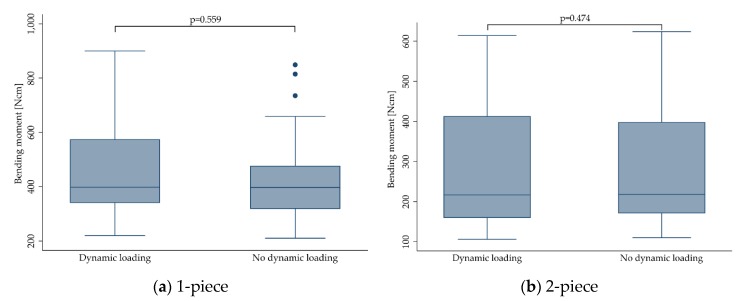
Boxplots showing the bending moment at the time point of fracture depending on dynamic loading (Yes: Implants were subjected to dynamic loading, No: Implants were not dynamically loaded) for 1- (**a**) and 2-piece (**b**) zirconia implants. Whiskers are used to represent all samples lying within 1.5 times the interquartile range. Dots represent outliers. Detailed data can be found in Table 3 and Table 4.

**Figure 10:**
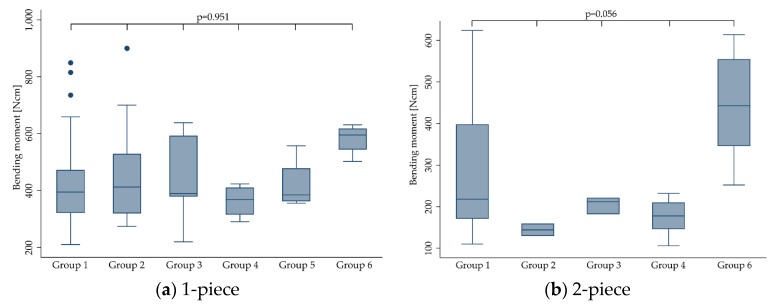
Boxplots showing the bending moment at the time point of fracture depending on dynamic loading conditions respecting the applied load and amount of cycles (as categorized in Section 2.7) for 1- (**a**) and 2-piece (**b**) zirconia implants. Whiskers are used to represent all samples lying within 1.5 times the interquartile range. Dots represent outliers. Detailed data can be found in Table 3 and Table 4. No 2-piece implants were allocated to group 5.

The effect of dynamic loading was evaluated from different perspectives. The simplest one assigned the included implants to two categories subjected to either no dynamic loading procedure (“No”) or those being subjected to dynamic loading (“Yes”; Figure 9, Table 4). Furthermore, the effect of dynamic loading was evaluated regarding the dynamically “applied load”, ranging from 45 [30] up to more than 500 N [17], or regarding the “amount of cycles” ranging from 1.2 [12,16,18,22,28,29,30] to 10 million [13,20,21,26] loading cycles. Finally, a combination of “applied load” and “amount of cycles” was used to from six groups, as mentioned in Section 2.7 (Figure 10).

When pooling the extracted data for 1- and 2-piece implants, dynamic loading did not affect the fracture resistance (dynamically-loaded implants showed a mean bending moment at the time point of fracture of 389.4 ± 169.2 Ncm compared to 383.2 ± 166.3 Ncm calculated for non-loaded implants (*p* = 0.410)). This did not change when evaluating 1- and 2-piece implants separately (*p* > 0.474). Solely the category “applied load” was close to statistical significance (*p* = 0.05). However, none of the multiple pairwise comparisons comparing different dynamically applied loads showed a statistically significant difference (*p* > 0.07). When solely evaluating 2-piece implants, “amount of cycles” significantly affected the fracture resistance (*p* < 0.0001), whereas “applied load” (*p* = 0.202) and groups 1–6 respecting the applied load and the amount of cycles (*p* = 0.056) did not affect the outcome.

## 4. Discussion

**Figure 11:**
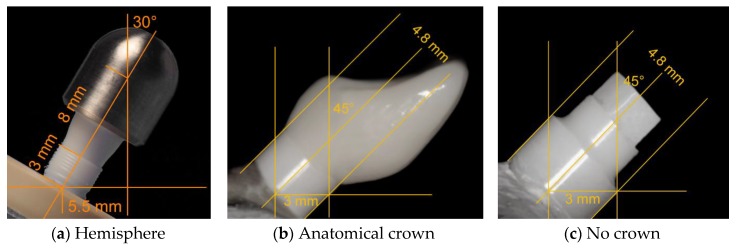
Exemplary schemes of embedded implants according to ISO 14801 (**a**) [21], equipped with an anatomically shaped incisor crown (**b**) or without any restorative supply (**c**) [20]. When embedding the samples according to ISO 14801, the lever arm measures 5.5 mm. In the latter two cases, the lever arm needs to be individually calculated and reported.

The present systematic review and meta-analysis included the data of 17 studies and two unpublished datasets. To be finally able to compare the outcomes of the included data, it was necessary to extract or calculate the bending moment at the time point of implant fracture [Ncm], since the mostly reported fracture load values [N] do not respect the leverage (length of the lever arm) and are therefore, if not considering a rigorously standardized embedding procedure as described in ISO 14801, not comparable to each other. Of the included 19 investigations/datasets, three studies reported the bending moment individually calculated for each included implant [13,20,22], whereas six studies [15,17,21,24,25,26] and the two included unpublished datasets fully respected ISO 14801 for embedding. Fully respecting this ISO implies the fixation of the endosseous part in a rigid clamping device or embedding in a material with a modulus of elasticity higher than 3 GPa. Moreover, the embedding/clamping level should respect a distance of 3.0 ± 0.5 mm apically from the nominal bone level, as specified in the manufacturer’s instructions for use. Furthermore, implant abutments need to be equipped with a non-anatomical hemisphere designed to realize a distance of l = 11.0 ± 0.5 mm from the center of the hemisphere to the embedding level (Figure 11).

When loading such samples with an angle of α = 30° to the vertical, the lever arm (y) or bending moment (M) for this configuration can be calculated with the reported fracture load (F) by using Equation (1).

(1) M=y·F=sin α·l·F

This results in y = 0.55 cm when embedding according to ISO 14801. For the aforementioned publications/datasets fully respecting ISO 14801 for embedding and reporting the fracture load values [N], the bending moment was therefore calculated by multiplying the fracture load with 0.55. Interestingly, some of the included investigations reported embedding according to ISO 14801, but solely adopted the embedding level (simulation of a bony recession of 3 mm), and sometimes the angulation (30°), but did not use a loading hemisphere, finally resulting in a lever arm different to 5.5 mm, as proposed by the ISO standard [16,19,27,28]. In most cases, anatomical crowns (maxillary premolars or incisors) made from ceramic materials were used instead of the hemisphere, finally resulting in altered lever arms and loading conditions. In the investigations of one group, the crown design and embedding procedure were described in detail (l, α and F were reported), allowing us to calculate y and M [19,27,28]. To calculate the bending moment for the remaining studies, authors needed to provide the necessary data upon request or standardized photographs provided in the publications, or by the authors needed to allow the approximation of the lever arm by using an image analysis software (ImageJ, National Institutes of Health, Bethesda, MD, USA) [12,16,18,29,30]. In order to be able to compare the outcome of preclinical studies evaluating the fracture resistance of dental implants, it is therefore recommended to either fully adopt an ISO standard for the embedding procedure or to provide the bending moment additionally to the fracture load. Considering different lever arms due to different embedding procedures for the implants included in this systematic review and meta-analysis, one needs to keep in mind that dynamic loading prior to static loading to fracture can result in altered fatigue, even if the applied load was the same.

The heterogeneity of the included samples comprising a mixture of market-available products (finally sterilized and incorporating a micro-roughened surface) [15,16,22,24,26] but also prototype implants (e.g., with or without any surface post-processing) [13,19,21,25,28,30] represents a major limitation of the present systematic review and meta-analysis. However, it was shown that, for example, surface modifications like micro-roughening to enhance osseointegration or steam-sterilization can significantly compromise fracture strength and ageing kinetics [31,32].

Another shortcoming of this systematic review presents the fact that of the 19 included datasets, more than half (nine published and two unpublished studies) were at least partially authored by the collaborates of the current paper. This might be considered a reasonable risk of bias. However, the present review was conducted according to standardized guidelines, and the available literature was systematically screened on the basis of predefined search terms and inclusion criteria. Modifying the search strategy, outcome measure or inclusion criteria in consequence of unexpected or homogeneously authored findings would likewise present a source of bias.

Regarding the treatments, the included samples have been subjected to prior to loading, and six groups (representing different categories of loading conditions as indicated in Section 2.7) have been evaluated for heterogeneity of the outcome. As a result, no heterogeneity of the bending moments for groups 1–6 was found (*p* = 0.612). This did not change when stratifying the implants according to their design (1-piece: *p* = 0.951; 2-piece: *p* = 0.056). Therefore, it was decided to pool the data of all groups for any further calculations, and yet still, one can hardly generalize the present findings and apply them to a specific zirconia implant system.

No statistically significant influence of hydrothermal aging on the fracture resistance of zirconia implants was calculated in the present review. It is important to note that aging or so-called low-temperature degradation (LTD) can, depending upon the sample quality and surface conditions, result in both increased [21,25] and decreased [33] fracture load. This might be explained by the following: Assuming a zirconia sample surface with various process-related defects/impurities, the largest defects/impurities are thought to act as “locus minoris resistentiae”, and can thereby be considered representative for the fracture resistance of this sample. Increased fracture load of such zirconia samples after a hydrothermal aging procedure is thought to be attributed to a transformed layer at the sample surface, inducing a compressive stress on the surface, tending to close a potential advancing crack at such existing defects/impurities located on the surface. This phenomenon is liable to cause an increase in the strength of the material, and was described for the first time three decades ago [34]. On the other side, at some point when the degradation process penetrates deeper into the material, the contribution from the aging may instead cause the strength of the same sample to be decreased, since once transformed to the monoclinic, zirconia grains cannot exhibit stress-induced phase transformation toughening anymore [33]. As an example, in the included investigation of Monzavi and co-workers [15] the effect of artificial aging on the mechanical resistance and micromechanical properties of commercially- and noncommercially-available zirconia dental implants was evaluated. In this study, the bending moment was significantly increased after aging for three of six groups, whereas two groups showed no influence of the aging procedure, and one group was negatively affected in terms of fracture resistance by the treatment [15]. When pooling the outcomes of the included studies showing positive, negative or no effects of LTD on the fracture resistance of zirconia implants in one dataset, as happened in the present meta-analyses, no effect of hydrothermal aging on the bending moment at the time point of fracture was calculated (*p* > 0.446). This, however, might be misleading, since several of the included studies indeed showed that aging can significantly affect the fracture resistance. However, due to the explanation given at the beginning of this paragraph, both in a negative or positive way. Therefore, missing significance, as calculated for pooled data in this review, should not be interpreted as an argument to refrain from aging tests of a zirconia implant system prior to market release. Therefore, pooling the data from different studies using the different conditions of thermal aging needs to be considered a limitation of the present review. It is discussed in the literature that the present amount of transformation to the monoclinic on the surface of as-delivered zirconia implants can be decisive for the ongoing fracture resistance after further hydrothermal aging procedures. In detail, implants showing no or very limited transformation to the monoclinic when released to the market (e.g., due to final temperature annealing [35] or manufacturing by ceramic injection-molding [21,25]) were observed to be less fracture-resistant in the original as-delivered state, but significantly gained fracture resistance due to increasing compressive stress at the sample surface after transformation to the monoclinic occurred. In contrast, samples already revealing a transformed layer of several micrometers (e.g., due to subtractive manufacturing or post-processing steps like sandblasting in order to roughen the surface to enhance osseointegration [26]) mostly do not benefit from further aging by means of an increased fracture resistance. Besides the amount of already transformed grains, implant surface topography showed to have a significant impact on aging susceptibility and its impact on fracture resistance [32,36]. As an example, implants structured with porous or alveolar surfaces were more likely to be negatively affected by aging procedures due to interconnected porosities in the surface layer, offering a path for the transformation to start at every surface accessible by water [25]. Finally, a layer structured in this way can be transformed in a shorter period of time.

Of the implants included in the present investigation, 209 of 731 were restored with anatomically-shaped crowns [16,19,20,27,28,29,30]. Most of these crowns were designed as maxillary central incisors, and were manufactured from: lithium disilicate [20], veneered [29] or monolithic [19,27,28] zirconia, or porcelain fused to metal [30]. Another included study restored the implants with maxillary first premolar restorations made from lithium disilicate [16], whereas Joda and collaborates restored the implants with non-anatomical hemispheres likewise made from lithium disilicate [24].

Most of the included studies not restoring the implants with anatomically-shaped crowns were conducted by adopting ISO 14801. According to this standard, the loading force shall be applied to the hemispherical loading surface, by a loading device with a plane surface normal to the loading direction of the machine, without additional horizontal loading forces. In contrast, especially incisor crowns present an inclined plane when loaded during the dynamic and finally static loading procedure, resulting in an increased shear force. Additionally, some investigations applied horizontal forces during the dynamic loading procedure (as it happens in the oral cavity), causing further fatigue of the sample [20,29,30]. Therefore, not the restoration itself, but the altered investigational setup, resulting in increased shear forces and fatigue during static loading, and in some cases, precedent chewing simulation might be considered responsible for decreased fracture resistance. Nonetheless, this finding should be taken into account when drafting international standards in order to guarantee clinical safety, since the anatomical reconstruction of zirconia oral implants and horizontal shear forces during loading represent clinical reality. Regarding the nature or location of failure, 1-piece implants mostly fractured at the embedding level or slightly below, with crack initiation on the tensile side of the implant. As described in the included studies, it seems that the fracture mode was not affected by crown supply. In 2-piece implants, fracture modes were generally observed to be highly heterogeneous, depending on the mode of assembly and the materials used.

When it comes to clinical reality, the fracture resistance of a zirconia implant should finally withstand the maximum voluntary bite forces of the patients. Nonetheless, one cannot find the definition of any indication specific (e.g., for implants installed in anterior or posterior regions) minimum value for the fracture strength of a zirconia implant in ISO 14801. This, as an example, is provided in detail in ISO 6872 for ceramic materials used for reconstructions (e.g., crowns, bridges) in dentistry [37]. Taking the highest bending moment measured in vivo (95 Ncm) with the help of strain gauge abutments into account [38], and applying a safety buffer of 100%, one might consider a minimum fracture resistance of 200 Ncm sufficient to guarantee clinical safety. When applying this requirement to the included studies, mostly 2-piece prototype implants and implants with a reduced diameter (≤ 3.3 mm) did not meet this demand [19,24,27,28,30].

Of the zirconia implants included in the present investigation, 577 were manufactured from Y-TZP and 154 from ATZ. Overall, implant stability was significantly affected by the material, in favor of ATZ (*p* = 0.002). When evaluating 1- and 2-piece implants separately, however, only 1-piece implants made from ATZ performed better (*p* = 0.001), whereas 2-piece implants performed the same, regardless of the material selection (*p* = 0.282). This might be explained by the fact that 1-piece zirconia implants or even, as an example, 2-piece titanium implants are mostly made from one single material (in the case of titanium: the implant, the abutment and the abutment screw are mostly fabricated from titanium). In contrast, most of the available 2-piece zirconia systems represent a multi-material complex comprising at least two or sometimes even three different materials. In some cases, only the implant body is manufactured from zirconia, whereas the screw (e.g., titanium or PEEK) and/or abutment (e.g., glass-fiber or polyetherketoneketone/PEKK) might be manufactured from different materials revealing different aging or degradation behavior during treatments (hydrothermal aging, dynamic loading), precedent to final static loading to fracture. To date, sound correlations to approximate intraoral aging conditions in an accelerated way in the dental laboratory are mostly available for zirconia ceramics, but missing for screw and abutment materials prone to degradation in aqueous environments, like e.g., polyetherketones [39,40]. In consequence, no standardized testing procedures were proposed to the present date, sufficiently evaluating multi-material, 2-piece implants regarding their fracture resistance, and individually respecting the degradation behavior of several included components. Regrettably, the sample size and heterogeneity of the extracted data gathered from 2-piece implants included in the present review did not allow for the statistical evaluation of a potential impact of the screw or abutment material on the fracture resistance of 2-piece zirconia implants. In one of the included studies, the aim was to measure the abutment rotation and fracture load of 2-piece zirconia implants screwed with three different abutment screw materials [16]. Implants and abutments of the included system were assembled with screws made from gold, titanium and PEEK.

As a result, no significant differences were found for these three materials, even if PEEK screws showed inferior results. When choosing PEEK as an abutment screw material, the incorporation of continuous carbon fibers proved to positively affect the maximum tensile strength of the screw [41]. However, a strengthening effect on the entire implant-abutment complex in case of zirconia implants still needs to be evidenced. In one of the included studies [26], a 2-piece ATZ implant system assembled with a carbon-fiber-reinforced abutment screw showed to be non-inferior compared to a market-established 2-piece titanium implant of a highly comparable design regarding its fracture resistance.

## 5. Conclusions

The null hypothesis of the present review, supposing no distinction between treatments and features in relation to bending moment when statically loading a zirconia implant to fracture, needs to be partially rejected. The focused question can be answered as follows: In general, 1-piece implants can be considered more fracture resistant than 2-piece implants, even if some of the included studies showed very promising results for 2-piece zirconia implants. When focusing on 1-piece implants, implants made from ATZ are more fracture resistant than implants made from Y-TZP. Due to its negative impact on fracture resistance, abutment preparation of 1-piece zirconia implants should be avoided. When drafting international standards to guarantee clinical safety, one should keep in mind that the loading of anatomically shaped crowns might result in the decreased fracture resistance of zirconia implants compared to non-anatomical loading hemispheres, as mentioned in ISO 14801. Further research is needed to define adequate hydrothermal aging and dynamic loading conditions for 2-piece ceramic implants, nowadays mostly comprising a multi-material complex.

## Figures and Tables

**Table 1:** A total of 731 one- and two-piece implants made from yttria-stabilized tetragonal zirconia polycrystal (Y-TZP) and alumina-toughened zirconia (ATZ), extracted from 17 studies and two unpublished datasets, subjected to different dynamic loading and thermal aging conditions prior to static loading to fracture, were finally included in meta-analyses.

First Author	Year	Ref.	*n*	Material	Pieces	Loading Cycles (×10^6^)	Thermal Aging
Andreiotelli	2009	[29]	88	Y-TZP	1	0, 1.2	TC, none
Kohal	2009	[30]	32	Y-TZP	2	0, 1.2	TC, none
Kohal	2010	[18]	72	ATZ, Y-TZP	1	0, 1.2, 5	TC, none
Kohal	2011	[12]	48	Y-TZP	1	0, 1.2, 5	TC, none
Rosentritt	2014	[28]	36	Y-TZP	1, 2	1.2	TC
Kohal	2015	[20]	48	Y-TZP	1	0, 5, 10	TC, none
Sanon	2015	[25]	30	Y-TZP	1	0	HT
Spies	2015	[22]	48	ATZ	1	0, 1.2, 5	TC, none
Kammermeier	2016	[27]	30	Y-TZP	1, 2	0, 3.6	TC, none
Preis	2016	[19]	32	ATZ, Y-TZP	2	1	TC, none
Spies	2016	[13]	48	ATZ, Y-TZP	1, 2	0, 10	HT, none
Joda	2017	[24]	11	ATZ	2	0	none
Spies	2017	[21]	28	Y-TZP	2	0, 10	HT, none
Ding	2018	[17]	29	Y-TZP	1	0, 5	none
Spies	2018	[26]	14	ATZ	2	0, 10	HT, none
Monzavi	2019	[15]	60	Y-TZP	1	0	HT, none
Stimmelmayr	2019	[16]	36	Y-TZP	2	1.2	TC
Kohal	2020	*	28	Y-TZP	1, 2	0, 10	HT, none
Zhang	2020	*	13	Y-TZP	2	0, 10	HT, none

* Unpublished data, Ref. = Reference, *n* = total number of included implants, TC = thermal cycling, HT = high temperature.

**Table 2:** Groups 1–6 (as indicated in 2.7) were tested for heterogeneity regarding the outcome.

Groups	Overall	1	2	3	4	5	6
Effect ^1^	395.27	407.42	397.99	262.17	400.73	579.96	448.94
*95% CI*	*330.2–460.3*	*338.4–476.4*	*272.1–523.9*	*195.0–329.3*	*249.2–552.2*	*521.8–638.0*	*373.7–524.1*
Intra ^2^	103.57	110.07	74.58	100.30	150.33	46.64	57.59
*95% CI*	*89.4–120.0*	*89.3–135.7*	*42.4–131.0*	*61.4–163.8*	*95.8–235.8*	*18.2–119.7*	*28.5–116.4*
Inter ^3^	126.06	126.92	133.58	$1.670\times e^{-15}$	146.81	$4.690\times e^{-18}$	77.72
*95% CI*	*86.5–183.8*	*82.1–196.2*	*65.5–272.3*	−∞–+∞	*59.0–365.5*	−∞–+∞	*33.9–178.2*

^1^ Mean bending moment [Ncm], ^2^ Standard deviation/variation within included studies, ^3^ Standard deviation/variation in between included studies.

**Table 3:** Calculated mean bending moment (in Ncm) and standard deviation depending on the implant design, several covariables and treatments.

	Overall ^1^			1-Piece			2-Piece		
	*n*	Mean	SD	*n*	Mean	SD	*n*	Mean	SD
**Overall**	*731*	386.4	167.6	*495*	431.9	151.0	*236*	291.7	162.4
**Material**									
*Y-TZP*	*577*	378.7	160.1	*383*	422.2	143.4	*194*	284.3	155.7
*ATZ*	*154*	418.7	106.0	*112*	475.8	180.7	*42*	318.6	194.0
**Manufacturing**									
*Subtractive*	*591* ^2^	397.5	177.4	*417*	457.4	154.4	*174*	260.1	149.6
*Injection molded*	*120* ^2^	364.8	116.7	*70*	329.4	73.7	*50*	426.8	154.4
**Implant diameter**									
*3.0–3.3 mm*	*15*	207.2	14.3	*9*	215.0	6.7	*6*	191.6	-
*3.8–4.4 mm*	*675*	394.9	170.4	*463*	441.3	152.7	*212*	293.6	165.0
*4.5–5.0 mm*	*41*	349.4	125.4	*23*	388.0	59.4	*18*	301.2	178.0
**Anatomical crown supply**									
*Yes*	*209*	237.5	96.6	*74*	327.0	65.4	*135*	186.9	71.4
*No*	*522*	455.2	147.7	*421*	453.2	154.8	*101*	463.9	114.2
**Abutment preparation**									
*Yes*	*-*	-	-	*112*	411.3	126.2	*-*	-	-
*No*	*-*	-	-	*383*	436.5	156.5	*-*	-	-
**Implant-Abutment-Connection**									
*Screw-retained*	*-*	-	-	*-*	-	-	*159*	327.5	179.0
*Bonded*	*-*	-	-	*-*	-	-	*77*	217.0	86.0
**Thermal aging**									
*Thermal cycling*	*310*	355.5	171.7	*218*	426.5	149.4	*92*	174.6	41.1
*High temperature*	*124*	392.9	115.9	*75*	362.6	96.4	*49*	453.4	135.1
*None*	*297*	406.2	180.4	*202*	464.0	163.2	*95*	299.9	164.6
**Dynamic loading**									
*Yes*	*391*	389.4	169.2	*250*	447.7	146.6	*141*	279.2	156.5
*No/Group 1*	*340*	383.2	166.3	*245*	417.7	153.6	*95*	303.5	171.2
*Group 2*	*86*	258.1	111.5	*66*	362.6	59.4	*20*	174.4	50.2
*Group 3*	*76*	394.7	211.2	*40*	457.3	188.1	*36*	144.4	15.0
*Group 4*	*132*	379.6	159.7	*96*	437.8	140.5	*36*	205.1	20.5
*Group 5*	*17*	580.8	55.7	*17*	580.8	55.7	*-*	-	-
*Group 6*	*80*	435.1	108.3	*31*	420.2	93.0	*49*	443.6	122.5

*n* = number of included implants, SD = standard deviation, ^1^ 1- and 2-piece implants pooled together, ^2^ the authors of one included study could not provide the manufacturing mode for all included implants [28].

**Table 4:** Significance (linear mixed models (LMMs), level of significance *p* < 0.05) was calculated for differences regarding the implant design, different covariables and treatments.

		Significance (*p*)		
Parameter	Options	Overall ^1^	1-Piece	2-Piece
Implant design	1-piece, 2-piece	0.004	-	-
Material	Y-TZP, ATZ	0.002	0.001	0.282
Manufacturing	Subtractive, injection-molded	0.749	0.076	0.095
Implant diameter	Range: 3.3–5.0 mm	0.327	0.273	0.191
Anatomical crown	Yes/No	<0.0001	0.080	<0.0001
Abutment preparation	Yes/No	-	<0.0001	-
Connection type	Screw-retained, bonded	-	-	0.584
Thermal aging	TC, HT, none	0.446	0.538	0.776
Dynamic loading	Yes/No	0.410	0.559	0.474
	Applied load [range: 50–500 N]	0.050	0.181	0.202
	Amount of cycles [range: 1–10 × 10^6^]	0.238	0.971	<0.0001
	Groups 1–6 [as indicated in 2.7]	0.612	0.951	0.056
Angulation	Range: 30–45°	0.215	0.671	0.003
Crosshead speed	Range: 0.5–10 mm/s	0.261	0.562	<0.0001

^1^ 1- and 2-piece implants pooled together, TC = thermal cycling, HT = high temperature.

## Appendix A

**Table A1:** Articles excluded after screening of the full-texts.

First Author	Year	Ref.	Reason for Exclusion
Young	1972	[42]	Narrative review
Kohal	2006	[43]	Root analogue implants
Silva	2009	[44]	No static loading to fracture (step-stress fatigue)
Silva	2011	[45]	No static loading to fracture (pendulum impact tester)
Van Dooren	2012	[46]	Narrative review and case report
Iijima	2013	[47]	No evaluation of dental implants (discs)
Mobilio	2013	[48]	No static loading to fracture (strain measurements), <5 samples
Sanon	2013	[36]	No static loading to fracture (step-stress fatigue)
Cattani-Lorente	2014	[31]	No evaluation of dental implants (bar-shaped samples)
Rohr	2015	[49]	Fracture on the restoration level
Kamel	2017	[50]	Calculation of bending moment not possible, no author response
Karl	2017	[51]	No static loading to fracture (insertion torque measurements)
Korabi	2017	[52]	No static loading to fracture (finite element analysis)
Monzavi	2017	[53]	No static loading to fracture (accelerated aging only)
Zietz	2017	[54]	No zirconia implants
Baumgart	2018	[55]	No static loading to fracture
Rohr	2018	[56]	Fracture on the restoration level
Zaugg	2018	[57]	Fracture on the restoration level
Faria	2019	[58]	No evaluation of dental implants (discs)
Nueesch	2019	[59]	Fracture on the restoration level
Rohr	2019	[60]	Fracture on the restoration level
Scherrer	2019	[61]	Fractographic analysis of clinically fractured zirconia implants
Siddiqui	2019	[62]	Cyclic fatigue w/o subsequent fracture loading
